# An Insight into Phytochemical, Pharmacological, and Nutritional Properties of *Arbutus unedo* L. from Morocco

**DOI:** 10.1155/2021/1794621

**Published:** 2021-11-22

**Authors:** Mohammed El Haouari, Najat Assem, Sushil Changan, Manoj Kumar, Sevgi Durna Daştan, Jovana Rajkovic, Yasaman Taheri, Javad Sharifi-Rad

**Affiliations:** ^1^Department of Biology and Earth Sciences, Laboratory of Pedagogical Engineering and Didactics of Sciences and Mathematics (IPDSM), Regional Center for Education Careers and Training (CRMEF Fès-Meknès), B.P: 1178 Taza-Gare, Taza, Morocco; ^2^Laboratory of Natural Substances, Pharmacology, Environment, Modeling, Health & Quality of Life (SNAMOPEQ), Polydisciplinary Faculty of Taza, Sidi Mohamed Ben Abdellah University of Fez, B.P.: 1223 Taza-Gare, Taza, Morocco; ^3^Division of Crop Physiology, Biochemistry and Post-Harvest Technology, ICAR—Central Potato Research Institute, Shimla 171001, India; ^4^Chemical and Biochemical Processing Division, ICAR–Central Institute for Research on Cotton Technology, Mumbai 400019, India; ^5^Department of Biology, Faculty of Science, Sivas Cumhuriyet University, Sivas 58140, Turkey; ^6^Beekeeping Development Application and Research Center, Sivas Cumhuriyet University, Sivas 58140, Turkey; ^7^Institute of Pharmacology, Clinical Pharmacology and Toxicology, Medical Faculty, University of Belgrade, Belgrade, Serbia; ^8^Department of Biosciences, College of Life and Environmental Sciences, University of Exeter, Exeter, UK; ^9^Phytochemistry Research Center, Shahid Beheshti University of Medical Sciences, Tehran, Iran; ^10^Facultad de Medicina, Universidad Del Azuay, Cuenca, Ecuador

## Abstract

*Arbutus unedo L*. (Ericaceae) is an evergreen shrub widely distributed in the Mediterranean region, particularly through the Moroccan forests. It is an important medicinal plant of great scientific interest due to its nutritional, pharmacological, and chemical properties. The objective of this review is to provide insights into traditional medicinal uses and phytochemical and pharmacological properties of *A. unedo* from Morocco. In Morocco, the plant has been used as a traditional medicine to treat several pathological conditions. Many phytochemical compounds have been reported in the plant, of which vitamins, carotenoids, flavonoids, polyphenols, tannins, and their derivatives are the most prevalent. Leaves and fruits of *A. unedo* contain the most significant number of phytochemicals among the species. Furthermore, researchers have demonstrated that *A. unedo* exhibited antioxidant, anticancer, antibacterial, antidiabetic, antiaggregant, and antihypertensive activities due to the presence of many biochemical compounds with health-promoting properties. According to different toxicity tests, the use of *A. unedo* is devoid of any significant side effects and/or toxicity. Despite its nutraceutical and health-promoting properties, Moroccan *A. unedo* remains underexploited mainly, and most of its traditional uses have not yet undergone scientific evidence-based research; therefore, improved knowledge about the potential value of the plant would allow understanding of its biological activity based on its phytochemical compounds that may contribute to the species preservation and valorization.

## 1. Introduction

During recent years, there has been an increasing reliance on phytonutrients and medicinal plants, due to their wide range significance in human health, food industry, and human nutrition [1–4]. According to the World Health Organization (WHO), 80% of the world's population depends on traditional medicine for primary health care, especially in Asian and African countries [5]. In fact, various reports from epidemiological studies have consistently shown an inverse association between regular consumption of fruits and vegetables and the risk of cardiovascular diseases (CVD) and certain types of cancer [6–10]. Furthermore, it has been reported that herbal remedies have fewer side effects and are better tolerated by patients than synthetic medications [11]. In addition, the WHO has recognized that traditional medicine is an important component in health care and has been encouraging research on development involving medicinal plants [12]. The health beneficial properties of medicinal plants are in part attributed to the antioxidant activity of their phytochemical compounds, such as phenols, flavonoids, tannins, alkaloids, carotenoids, vitamins, and endogenous metabolites [13–15].

*Arbutus unedo* (*A. unedo*), the strawberry tree (Ericaceae), is widely distributed in the Mediterranean region. It can be also found in Canary Islands and western Asia, where climate is adequate to its development [16, 17]. This fruit tree species is known by different vernacular names, such as sasnou and bakhanou in Morocco, madroño in Spain, arbousier in France, Koumaria in Greek, and corbezzolo in Italy [18].

**Figure 1:**
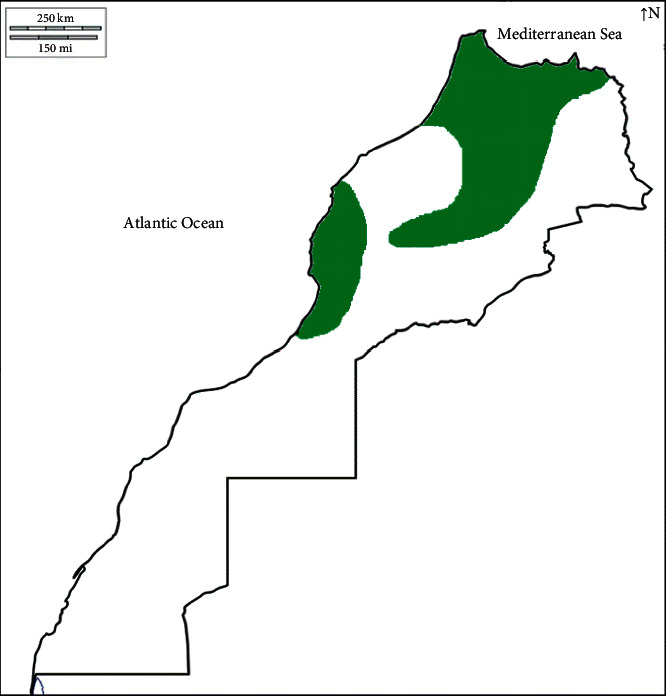
Approximate distribution of *Arbutus unedo* L. throughout Morocco.

In Morocco, strawberry trees grow wild in different bioclimatic regions extending from the subhumid to the semiarid regions (Figure 1). *A. unedo* has been extensively found in the region of the Northwest, Central Plateau, Pre-Rif and Western Rif, High and Middle Atlas, mainly associated with *Quercus ilex* and sometimes with *Pinus halepensis* and *Tetraclinis articulate.* A fragmented-like distribution is common in the Central, Souss plateau, and Northeastern regions. *A. unedo* shows strong resistance to hard environmental conditions and has the ability to regenerate after forest fires. This plant populations are widespread on all types of substrates but most often on siliceous and calcareous soils at altitudes varying from 150 to 1613 m. The average of annual temperature and rainfall varied from 12.4°C to 18.4°C and from 337 to 1115 mm, respectively [19].

Traditionally, the fruits, leaves, and roots of *A. unedo* are well-known and used by the Moroccan population as diuretic, astringent, antidiarrheal, antiasthmatic, antiinflammatory, antidiabetic, antihypertensive, against rheumatism and gastrointestinal and renal diseases [20–27]. However, all these uses remain more as traditional habits than having economical purposes.

A diversity of pharmacological properties is ascribed to *A. unedo*, such as astringent, depurative, anti-inflammatory, haemostatic, antitumor, antioxidant, antimicrobial, spasmolytic, and neuroprotective [28–40].

Furthermore, experimental investigations of our group have shown that *A. unedo* extracts exhibited different biological properties including antioxidant, platelet antiaggregant, vasorelaxant, antihypertensive, and antidiabetic activities [37, 41–49]. In addition, the plant has been shown to contain different phytoconstituents such as flavonoids, tannins, phenolic acids, organic acids, *α*-tocopherol, carotenoids, anthocyanins, triterpenoids, fatty acids, sterols, vitamin c, fibers, calcium (Ca), potassium (K), magnesium (Mg), phosphorus (P), and other bioactive compounds [37, 47, 50–55], which contribute to its various pharmacological and nutritional properties.

In Morocco, no economic importance was attributed to *A. unedo*, and the plant remains largely underexploited, in comparison to other Mediterranean countries, where the fruit of the *Arbutus* tree is highly sought after for its nutritional qualities [56–58]. Furthermore, *A. unedo* tree populations are severely destroyed due to deforestation and overcollecting. In addition, review publications related to *A. unedo* from other countries can be found in the literature; however, to the best of our knowledge, there are no comprehensive scientific reviews to cover all aspects of information about *A. unedo* grown in Morocco. Thus, an improved knowledge about the potential value of the plant could contribute to enhance the production, marketing, and consumption of *A. unedo* derived products, which may contribute to the species preservation and valorization. In this context, the aim of this work is to provide a comprehensive review concerning the ethnobotanical, pharmacological, nutritional, phytochemical, and toxicological properties of *A. unedo* grown spontaneously in Morocco, which may encourage interested researchers to conduct further investigations to evaluate the health-promising benefits and nutritional properties of Moroccan *A. unedo*, its active constituents, and their derivatives.

## 2. Search Strategy

Available information was collected from different scientific databases such as PubMed, Science direct, Springer, Web of Science, and Wiley using keywords: *Arbutus unedo* L., *A. unedo* L., strawberry tree, ethnobotanical, phytochemical and Pharmacological properties. Additional references were hand-searched. This review is limited to scientific research concerning the ethnobotanical, pharmacological, and phytochemical properties of *A. unedo* L. grown in Morocco.

## 3. Botanical Description

**Figure 2:**
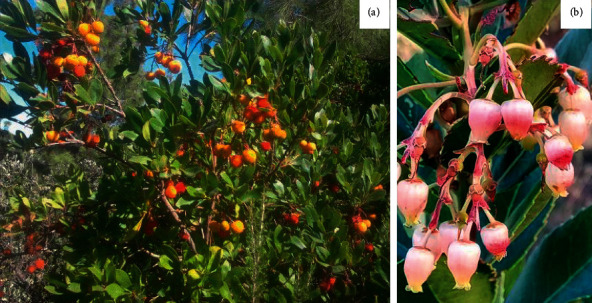
The whole plant (a) and flowers (b) of *Arbutus unedo* L.

*A. unedo* belongs to family Ericaceae that is in the major group Angiosperms (flowering plants). It is an evergreen shrub with erect and branched stems (Figure 2). The plant can reach a height of 12 meters, but it is normally a shrub between 1.5 and 3 meters tall [16]. It is an important ornamental bush due to its nice look and fragrance of the white flowers and the red fruit, which appear simultaneously in autumn and winter. The leaves are evergreen, entire, oblong-lanceolate, short-stalked, and coriaceous with a dark green color and finely serrated margin [59]. The flowers are hermaphrodites, fragrant, white, and like little bells, with 5 green sepals fused at the base, 5 united petals, and 10 stamens [18]. *A. unedo* fruit is a spherical berry about 1–2 cm in diameter, with a rough surface, green to yellow to bright red when fully ripe. The strawberry tree bears both flowers and fruits at varying degrees of maturity. This is due to the long ripening period of the fruits, which is more than one year [59].

## 4. Traditional Uses

In Morocco, different parts of *A. unedo* are used in folk medicine to treat various ailments (Table 1). A survey of medicinal plants in Oriental Morocco showed that *A. unedo* roots and leaves are widely used in the treatment of hypertension and diabetes [22]. Authors reported that the phytotherapy is widely adopted in northeastern Morocco. About 67.5% of total identified patients regularly utilized phytotherapy to treat their diseases. In another ethnobotanical survey realized in Northern Morocco, the leaves and the roots of the plant were used traditionally to treat diabetes, hypertension, and cardiac diseases [23]. Furthermore, the roots and cortex of the plant have been also used as a remedy for cholesterol-lowering, against digestive problems and CVD [60]. In another ethnobotanical study, El-Hilaly et al. reported that the leaves and fruits of *A. unedo* are orally administered in the form of decoction as well as raw by the population in the region of Taounate for the treatment of renal diseases [24]. In Ouezzane (North-West of Morocco), the leaves of the plant have been used as decoction in the treatment of diabetes [61]. Other ethnobotanical surveys realized through Morocco revealed that the plant is also used by the population as stomachic, diuretic, astringent, antidiarrheal, antiasthmatic, and anti-inflammatory medicine, and against rheumatism [20, 25, 27]. There are numerous reports, which showed widespread utilization of *A. unedo* to heal various disease conditions in Morocco. Therefore, there is an urgent need to utilize *A. unedo* as an effective alternative to modern allopathy medicines to heal several diseases.

## 5. Phytoconstituents

**Figure 3:**
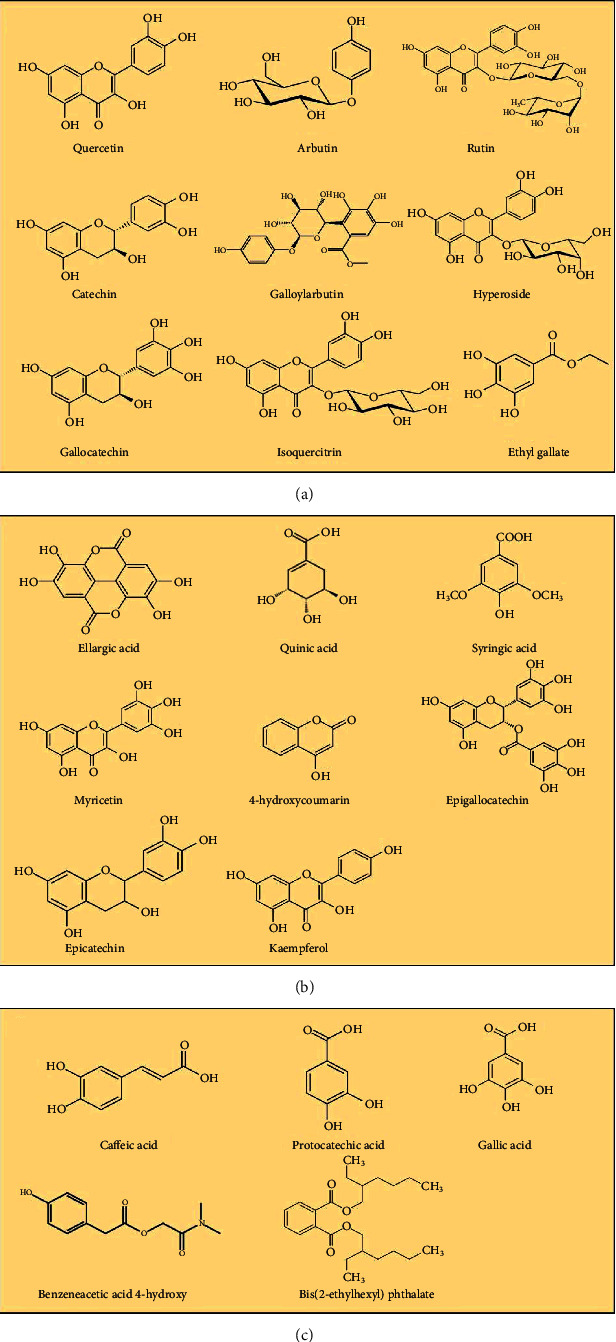
Structure of various bioactive compounds present in leaves, roots, and fruits of strawberry trees.

The plant is also an important source of phytochemical compounds; therefore, many bioactive compounds are isolated. It is revealed that genetic variability and environmental factors may affect the phytochemistry of *A. unedo*. Phytochemicals found in various parts of Moroccan *A. unedo* are given in Table 2, and the structures are shown in Figure 3.

### 5.1. Leaves

A number of phytochemical studies revealed that *A. unedo* leaves contain various classes of chemical compounds such as phenolic compounds, terpenoids, anthocyanins, flavonoids, and tannins. In a previous study, we have demonstrated the presence of genins and heterosidic flavonoids in the extracts of *A. unedo* leaves collected from Tazzeka Mountain (region of Taza) [37]. In another study, we have isolated tannins from *A. unedo* leaf extract [48]. Other phytochemical analysis reported that *A. unedo* leaves contain various polyphenolic compounds, such as epicatechin, catechin, and catechin gallate [47].

In a screening study, Mrabti et al. reported that *A. unedo* leaves from the Beni Mellal region contain tannins, flavonoids, and anthraquinones at high concentrations, and free quinones and terpenoids in lower concentrations. A study of Kachkoul et al. found that *A. unedo* leaf extracts from Taounate region contain polyphenols, flavonoids, flavonols, and anthocyanins [62]. The level of anthocyanins was low with regard to other compounds. The authors used ultraperformance liquid chromatography (UPLC-PDA-ESI-MS) to characterize the chemical compounds in the leaves. The UPLC profile indicated the presence of 22 compounds of gallic acid derivatives and flavonoids [62]. In another study, it has been reported that hydroalcoholic and aqueous extracts from *A. unedo* leaves contain tannins, polyphenols, and flavonoids [63]. Thus, the consumption of *A. unedo* leaves as raw or decoction would provide a sufficient amount of health-promoting phytochemicals.

### 5.2. Fruits

Little information is known about the chemical composition of Moroccan *A. unedo* fruits. Recently, Zitouni and coworkers [64] have analyzed the phenolic compounds in five strawberry tree fruits from different locations in Morocco and found that total phenols varied from 25.37 to 39.06 mg GAE/g D.W, while total flavonoid ranged from 3.30 to 7.07 mg GAE/g D.W. In total, 17 phenolic compounds have been identified in sampled cultivars, of which gallocatechol and catechin were the most abundant.

El Cadi et al. investigated the phytochemical profile of strawberry tree fruits grown in different locations of Northern Morocco (Achakar, Qsar Kbir and Chaoun-Qalaa). Results indicate that the chemical composition of the fruits varied between the three regions. Indeed, fruits from Achakar showed a high amount of polyphenols and flavonoids (127.5 ± 7.14 mg GAE/g (w/w) (D.W) and 105.9 ± 3.2 mg QE/g (w/w) (D.W), respectively), while berries from Qsar Kbir presented a high content of anthocyanins and tannins (1.48 ± 0.09 mg Pg-3-glu/g (w/w) (D.W) and 1.22 ± 0.1 mg EC/g (w/w) (D.W), respectively). In addition, chemical analysis of the three fruits using HPLC-DAD-ESI/MS revealed the presence of 75 chemical compounds, including hydroxybenzoic acids, hydroxycinnamic acids, flavone, flavan-3-ols, flavonols, and dihydroflavonols [65]. It was suggested that the variability observed in phytochemical composition among the different fruit extracts could be due to the geographical location of the samples.

In another study, Zitouni et al. determined the content of the total phenolic compounds and flavonoids, condensed and hydrolyzable tannins, and anthocyanins in twelve genotypes of *A. unedo* fruits collected from different locations of Morocco. Results showed that total phenolic compounds and flavonoids varied from 22.63 ± 1.74 to 39.06 ± 2.44 mg GAE/g D.W and from 3.30 ± 0.60 to 8.62 ± 1.10 mg RE/g D.W, respectively, while anthocyanins varied from 0.12 ± 0.06 to 0.66 ± 0.15 mg cya-3-glu/100 g D.W; however, condensed tannins and hydrolyzable tannins ranged between 10.41 ± 1.07–16.08 ± 1.50 mg TAE/g D.W and 4.08 ± 2.43–6.34 ± 3.47 TAE/g D.W, respectively. Using the HPLC assay, the authors also identified 17 phenolic compounds in the studied fruits, of which gallocatechol and catechin were the most abundant [66]. It was concluded that genetic factors seem to be responsible for the chemical profile of the analyzed fruits. The significant amounts of phytochemicals in *A. unedo* fruits confirm the nutritional and medicinal value of the plant.

### 5.3. Roots

**Figure 4:**
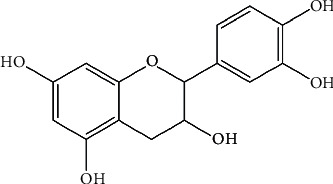
Chemical structure of catechin isolated from *A. unedo* roots [50].

There are few studies that have focused on the chemical composition of the Moroccan *A. unedo* roots. Using Zippertex technology, Mrabti et al. isolated catechin (the therapeutic active compound) from *A. unedo* roots (Figure 4). The chemical structure of this compound was characterized by MS and NMR (^1^H and ^13^C) analyses [50]. In addition, similar to leaves, the roots of *A. unedo* were shown also to contain tannins, anthraquinones, and flavonoids [67]. Furthermore, scientific investigations are needed to unearth the phytochemical profile of *A. unedo* roots and its applications to treat several diseases.

## 6. Nutritional Characterization

The berries of *A. unedo* are popular in Morocco and consumed by the population as food since a long time, while the leaves and roots are commonly used for medicinal applications to treat ailments. The fruits have a sweet taste and contain a wide variety of molecules with an excellent nutritional quality including phenolic compounds, sugars, dietary fiber, *α*-tocopherol, proteins, unsaturated fatty acids, organic and phenolic acids, arbutine, vitamins, and carotenoids [56, 57, 68–71].

In Morocco, ripened fruits of *A. unedo* are eaten as a remedy for some health problems such as gastrointestinal, infective, and urological diseases [21, 24]. Their consumption provides the body with a significant amount of nutrients, vitamins, minerals, sugars, and other bioactive compounds with health beneficial effects. The fruits are not consumed very often, but they can be enjoyed when eaten in moderate quantities. However, if taken in excess, on fasting, they would be purgative. They are usually eaten in the field as fresh fruits and sometimes taken home for dessert or sold by rural people at the weekly markets or by the roadside in small drum or reed baskets. Often this product constitutes a complement to rural income.

A study by El Cadi et al. demonstrated that *A. unedo* fruits collected from different parts of Morocco are with high antioxidant capacity. It has been concluded that this fruit is a source of various potentially therapeutic compounds for the treatment of many diseases [65]. In addition, several studies reported that *A. unedo* leaves constitute valuable flavonoids and minerals, especially K, Ca, P, and Mg [47, 67]. It was suggested that the rich mineral and polyphenols content of *A. unedo* leaves can constitute an interesting addition to human diet and therapy.

Despite its high nutritional value, strawberry fruit valuation is still timid in Morocco and is limited to the production of honey, jams, and pastries by some cooperatives located in the Western Rif of Morocco [19].

## 7. Pharmacological Activities

**Figure 5:**
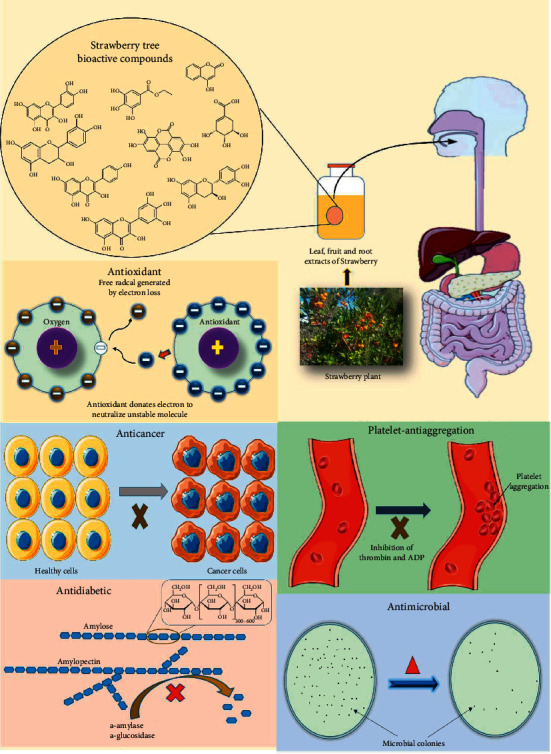
Representation of some pharmacological activities of *Arbutus unedo* L.

The documented pharmacological activities of Moroccan *A. unedo*, its extracts, and compounds isolated from this species are detailed below and are summarized in Table 3, and some of the pharmacological effects are illustrated in Figure 5.

### 7.1. Antidiabetic Activity

Type 2 diabetes mellitus (DM) is a complex syndrome characterized by chronic hyperglycemia as a consequence of a disorder of insulin secretion, insulin resistance/action, or combination of both of these factors. It is estimated that 25% of the world population is affected by this disease [72]. Furthermore, DM is considered as the major risk factor for CVD including heart ischemic disease, stroke, atherosclerosis, and heart failure [73]. Therefore, the identification of natural antidiabetic compounds with insignificant toxicity and no side effects is of great interest [74]. In this regard, several medicinal plants have been used traditionally for the treatment and/or prevention of DM [75].

The antidiabetic activity of *A. unedo* roots extract has been tested in rats using the oral glucose tolerance test (OGTT) and the intravenous glucose tolerance test (IVGTT). Oral administration (500 mg/Kg b.w) of the extract to rats submitted to OGTT produced a significant decrease in glycaemia after glucose overload. This effect was not confirmed by the IVGTT. Furthermore, *A. unedo* extract exhibited significant reduction of intestinal glucose absorption, which may justify in part the reduction of glycaemia observed in the OGTT model [41].

In a pre-clinical study, it has been demonstrated that the oral administration of water extracts from *A. unedo* roots (400 mg/L, drink water) showed significant reduction of glycaemia in neonatal streptozotocin (n-stz)-induced diabetic rats under chronic treatment [46]. Furthermore, the authors reported that the combination of the plant extract with insulin enhanced the peripheral utilization of glucose and potentiated the activity of insulin, which may explain in part the hypoglycemic effect observed with the *in vivo* test [46]. It is concluded that *A. unedo* roots contain bioactive compounds, which contribute to their antidiabetic activity and validate their utilization in traditional medicine.

In another study, Mrabti et al. assessed the antidiabetic properties of *A. unedo* root extract *in vitro* and *in vivo*. In the *in vitro* test, results showed that *A. unedo* root extract exerted more potent inhibitory effect against *α*-glucosidase than acarbose (positive control) with IC_50_ (median inhibitory concentration) values of 94.81 ± 5.99 µg/mL and 199.53 ± 1.12 µg/mL respectively. In the *in vivo* antidiabetic study, it has been found that *A. unedo* extract (500 mg/Kg b.w) produced a significant decrease in glycaemia similar to that of metformin (positive control) in STZ–NA induced-diabetic mice after chronic oral administration for 4 weeks. In addition, treatment with the extract (500 mg/day/kg b.w) resulted in the restoration of the histological architecture of the islets of Langerhans in diabetic mice [76]. The possible action mechanism by which *A. unedo* exerts its hypoglycemic effect may be related to the pancreatic secretion of insulin from the existing beta cells or by its release from the bound form [77].

The effects of the root bark aqueous extract of *A. unedo* on intestinal glucose absorption was studied by Mrabti and coworkers *in vitro* and *in vivo*. Results showed that the tested extract (10 *μ*g/mL to 1 mg/mL) inhibited dose-dependent sodium-dependent glucose transport across isolated mouse jejunum with an IC_50_ value close to 216 *μ*g/mL. In addition, the *A. unedo* extract (2 g/kg/day) improved oral glucose tolerance and reduced body weight after chronic oral administration during 4 weeks in rats [78]. These results support the traditional use of *A. unedo* in the treatment of diabetes.

In another study, Mrabti and colleagues isolated catechin from *A. unedo* roots and evaluated its antidiabetic activity using the *α*-glucosidase test. Results showed that the isolated compound exerted antidiabetic activity through inhibition of *α*-glucosidase enzyme activity with an IC_50_ value of 87.55 ± 2.23 *µ*g/mL [50]. The inhibitory potential of catechin was greater than that of the positive control acarbose (IC_50_ = 199.53 ± 1.12 *µ*g/mL). The antidiabetic activity for root extract of *A. unedo* is very well established by various researchers. Therefore, there is need to explore the potential of *A. unedo* leaves to treat diabetes. However, there is no adequate studies on the effect of *A. unedo extracts* with respect to molecular aspects.

### 7.2. Antihypertensive and Vasorelaxant Activity

Hypertension is a major risk factor for CVD, such as coronary heart disease and stroke, the two leading causes of death among adults worldwide [79]. In addition, current antihypertensive agents have limited effectiveness and various side effects. Recently, various studies have been focused to find new drugs from medicinal plants with antihypertensive properties.

*A. unedo* has been tested for antihypertensive activity. Afkir et al. reported that the simultaneous oral treatment of rats with L-NG-nitroarginine methyl ester (L-NAME) and *A. unedo* leaf or root aqueous extract (250 mg/Kg/day) for 4 weeks inhibited hypertension development and reduced ventricular hypertrophy (root extract). Furthermore, the extracts normalized renal function, improved vascular reactivity, and baroreflex sensitivity in rats [42].

In another pre-clinical study, it has been reported that the chronic treatment of spontaneously hypertensive rats (SHR) with *A. unedo* root aqueous extracts (5, 50 and 250 mg/kg/24 h) delayed the development of hypertension but did not change the final level of blood pressure and heart rate. A diuretic effect was observed in the group receiving the highest dose (250 mg/kg/24 h) of *A. unedo* [49].

The vascular activity of *A. unedo* roots was also examined *in vitro* using isolated rat aorta. It has been found that the root aqueous extract (0.25 mg/mL) induced an endothelium-dependent vasodilation of the isolated rat aorta, and this effect was mainly attributed to the activation of the endothelial nitric oxide synthase (NOS) [43].

In another *in vitro* study, Legssyer and colleagues examined the vascular effect of aqueous extract from *A. unedo* leaves on the isolated rat aorta. Results showed that the *Arbutus* leaf extract (0.01 g/L) produced a strong endothelium-dependent vasorelaxant activity. A chromatographic fractionation of the methanolic extract of *A. unedo* leaves showed that this vasorelaxant activity was probably assigned to polyphenolic compounds, such as tannins and catechin gallate [47]. It was suggested that these findings may account for the antihypertensive property of *A. unedo* reported in folk medicine. Furthermore, studies are required to unravel the molecular mechanism of action of phytochemicals present in *A. unedo* extracts.

### 7.3. Antiaggregant Effect

Blood platelets play a crucial role in the primary hemostasis through the formation of a platelet clot. However, platelet hyperactivity is mainly involved in thrombosis and atherosclerosis [37, 80, 81]. Furthermore, the use of anti-platelet agents is often associated with various adverse effects including bleeding problem and gastric problems [82]. In this context, the development of anti-platelet drugs of natural origin is of great interest [2].

In a study, the platelet antiaggregant effect of *A. unedo* was investigated *in vitro* by a group of researchers using different extract preparations. In a screening study, it was demonstrated that *A. unedo* root extract from Tazekka Mountain inhibited *in vitro* platelet aggregation induced by thrombin or ADP in a dose-dependent manner [45]. In another investigation, it was reported that the leaf aqueous extract of the plant inhibited thrombin-induced rat platelet aggregation *in vitro* with an IC_50_ value of 1.8 g/l. Successive extraction of *A. unedo* leaves with solvents of increasing polarity (petroleum ether, dichloromethane, ethyl acetate, and methanol) revealed that ethyl acetate and the methanolic extracts were most active against platelet aggregation with IC_50_ values of 0.6 ± 0.05 and 0.7 ± 0.08 g/L, respectively [48]. In order to verify if the tannins were implicated in the obtained antiaggregant effects, these compounds were isolated from the methanolic extract, and their anti-platelet effect was evaluated *in vitro*. The obtained results indicated that tannins precipitated by caffeine induced an important antiaggregant effect, whereas the adsorption of these compounds by skin powder in the methanol extract reduced significantly its activity. Thus, the antiplatelet aggregation effect of the *A. unedo* leaf extract was mainly attributed to condensed tannins [48].

In another study, researchers examined the anti-platelet effect of flavonoids from *A. unedo* leaves *in vitro*. It has been found that genins (free flavonoids) hetrosidic flavonoids reduced significantly thrombin-induced platelet aggregation with IC50 values of 0.22 ± 0.03 and 0.36 ± 0.05 mg/ml, respectively. In addition, at 1 mg/ml, these compounds reduced significantly the initial rate of platelet aggregation by 97.8 ± 0.74% and 90.8 ± 1.55% for genins and heterosidic flavonoids, respectively [44]. Thus, these findings clearly demonstrate that the anti-platelet effect of *A. unedo* is mainly attributed to flavonoids.

In addition to our work on rat platelets, we have investigated the effects of flavonoids from *A. unedo* leaves on human platelets. It has been found that the aqueous extract and flavonoids from *A. unedo* leaves exerted anti-platelet effects in human platelets [37]. Furthermore, it was demonstrated that at 0.05 mg/mL, the tested compounds reduced thrombin-induced endogenous ROS production, as well as Ca^2+^ mobilization and protein tyrosine phosphorylation, two processes that have been revealed to be regulated by the redox potential in platelets [83, 84]. These findings are in agreement with the antiaggregant effect of *A. unedo* observed in rat platelets [45, 48], although, it should be noted that human platelets seems to be more sensitive to the extracts than rat platelets. It was concluded that *A. unedo* extracts exhibited anti-platelet activity by a mechanism implying the reduction of Ca^2+^ mobilization, ROS production, and protein tyrosine phosphorylation, which might support the traditional use of this plant in the treatment and/or prevention of CVD. However, further studies to understand its molecular mechanism and in-depth in vivo investigations may help to include *A. unedo* as an alternative to modern medicine.

### 7.4. Antioxidant Activity

Oxidative stress has been mainly implied in the development of chronic and degenerative diseases such as cancer, diabetes, and cardiovascular and neurological diseases [85]. Increased consumption of plant-based foods (fruits, vegetables, and nuts) has been reported to reduce and prevent damage caused by free radicals [86].

Numerous studies have evaluated the antioxidant activity of *A. unedo in vitro* and *in vivo*. Different compounds were isolated from the plant and were evaluated for their antioxidant potential. Recently, Zitouni and coworkers [64] evaluated *in vitro* the antioxidant properties of *A. unedo* fruits from different areas of Morocco using three antioxidant essays (1,1-diphenyl-2-picrylhydrazyl (DPPH), 2,2-azinobis-(3-ethylbenzothiazoline-6-sulfonic acid) (ABTS), and *β*-Carotene bleaching). Results revealed significant differences among sampled fruits. The average radical scavenging capacities were 3.33–21.08, 2.25–19.58, and 1.08–13 mg Ascorbic Acid Equivalent (AAE)/g d.w) for the DPPH, ABTS, and *β*-carotene bleaching assays, respectively. The authors suggested that the difference in results might be due to difference in varieties and/or growing region of the *A. unedo* samples.

In another recent study, Zitouni and colleagues assessed the antioxidant activity of phenolic, flavonoids, condensed tannins, hydrolyzable tannins and anthocyanins from twelve *A. unedo* fruits genotypes using ABTS. Significant variations were observed in the antioxidant activity among the studied extracts. The IC_50_ value (ABTS) of the tested compounds varied from 1.75 to 19.58 mg AAE/g d.w [66]. It is noticeable that the antioxidant activities of the tested compounds depend on the region and diversity of geographical environments.

Kachkoul and coworkers analyzed the antioxidant activity of aqueous and hydroalcoholic extracts from *A. unedo* leaves using DPPH and the ferric reducing/antioxidant power (FRAP) assays. It has been found that the hydroalcoholic extract exerted higher antioxidant capacity than the aqueous extract with IC_50_ values of 76.14 ± 0.91 *µ*g/mL for hydroalcoholic extract versus 202.64 ± 5.77 *µ*g/mL for aqueous extract using the DPPH assay, and 53.77 ± 0.81 *µ*g/mL for hydroalcoholic extract versus 236.86 ± 31.90 *µ*g/mL for aqueous extract using the FRAP test [62].

The antioxidant activity of *A. unedo* roots and leaves aqueous extracts was determined by Mrabti et al. *in vitro* and *in vivo*. The *in vitro* antioxidant activity was determined using the DPPH assay, while the *in vivo* activity was analyzed by dosage of malondialdehyde (MDA) and superoxide dismutase (SOD) in diabetic mice. The IC_50_ values for the *in vitro* antioxidant activity of the aqueous extract was 4.52 and 7.24 *µ*g/mL in roots and leaves, respectively [67]. In addition, there was no significant difference between *A. unedo* and metformin group (positive control) in all of the *in vivo* examined parameters (MDA and SOD) in liver and kidney of diabetic mice [67]. The high antioxidant potential of the roots is compatible with their higher content in tannins, anthraquinones, terpenoids, and flavonoids. These results suggest that *A. unedo* may strengthen the enzymatic antioxidant system and reduce tissue damage due to oxidative stress.

In another study, Bouyahya and coauthors assessed the antioxidant activity of methanolic, ethanolic, ethyl acetate and n-hexanic extract from *A. unedo* leaves using DPPH assay. It has been found that the plant extracts showed an antioxidant effect against DPPH radicals. In addition, n-hexane and methanol extracts were found to exert the highest radical scavenging activity with IC_50_ values of 73.73 *μ*g/mL and 95.25 *μ*g/mL, respectively [87]. The differences of the obtained results could be related to the type of solvents and the nature of phytochemical content of each extract.

In a recent study, El Cadi et al. determined the antioxidant capacity of *A. unedo* fruits from three locations of northern Morocco (Achakar, Qsar Kbir and Chaoun-Qalaa) using the DPPH assay. Results showed that all the samples have antioxidant activity (EC50 values between 1.37 ± 0.2 and 17.82 ± 0.12 mg/mL (w/v)). Higher antioxidant capacity was observed with samples extracted by MeOH:water. In addition, Chaoun-Qalaa fruits were found to exhibit the highest antioxidant activity, which is compatible with their higher total polyphenol and flavonoid content [65]. The authors concluded that *A. unedo* fruits contain various phytochemicals with high antioxidant capacity, and could be, therefore, used in pharmacological and nutritional research. The antioxidant potential of *A. unedo* extracts has been justified by several in vitro and in vivo investigations, still there is enough scope to study the effect of phytochemicals present in *A. unedo* on gene expression levels. Additionally, these investigations mainly used the whole crude extracts of *A. unedo* root and leaves instead of the bioactive compounds.

### 7.5. Antibacterial and Antiparasitic Activity

Antimicrobial agents play a major role against infectious diseases. However, the consumption of antibiotics can lead over time to the development of a more resistant bacteria [88]. Antimicrobial resistance makes antibiotics less effective, and infections become difficult to be treated. Thus, the need to find new and efficient antimicrobial agents is of great interest. In this sense, various medicinal herbs have been used due to their antimicrobial properties [89].

Bouyahya and coworkers determined the antimicrobial activity of methanolic, *n*-hexanic, ethyl acetate, and ethanolic extract from *A. unedo* leaves against *Escherichia coli* K12 MBLA, *Staphylococus aureus* CECT 976, *Listeria monocytogenes* Serovar 4b CECT 4032, and *Pseudomonas aeruginosa* IH using the agar well diffusion assay. A significant difference in activities was observed against the studied bacterial strains. The highest activity was obtained with the *n-*hexanic extract, particularly against *S. aureus* and *L. monocytogenes* (zones of inhibition varied from 34.42 ± 0.26 mm to 40 ± 0.19 mm) [87]. The inhibition was related to the presence of phenolic and flavonoid compounds.

In another study, the extra- and intracellular antimycobacterial activities of aqueous and ethanol extracts from *A. unedo* leaves were studied against the growth of three mycobacteria (*Mycobacterium bovis*, *Mycobacterium smegmatis*, and *Mycobacterium aurum*). Results showed that the tested extracts exhibited extracellular antimycobacterial activity against the growth of three *mycobacteria*. The minimum inhibitory concentration (MIC) of the ethanolic extract was 5.59 ± 0.69 mg/ml for *Mycobacterium aurum* A+ and 6.02 ± 0.76 mg/ml for *Mycobacterium smegmatis* MC2 and *Mycobacterium bovis* PPI. Moreover, ethanol extract used at 6.02 ± 0.76 mg/ml showed potent intracellular antimycobacterial activity against *M. smegmatis* MC2 located within the rat peritoneal macrophages [90]. The antimycobacterial activity of the plant could be assigned to a number of phytochemicals, such as phenolic compounds.

Leishmaniasis is a parasitic disease caused by *Leishmania* parasites, which are transmitted by phlebotomy insects. It constitutes a serious health problem worldwide, especially in Africa [91, 92]. Therefore, the search for antileishmanial drugs with no side effects is of great interest. In this sense, the antileishmanial activity of methanolic, *n*-hexanic, and ethanolic extract of four medicinal plants including *A. unedo* was examined by Bouyahya et al. against *Leishmania major*, *Leishmania tropica*, and *Leishmania infantum* using the MTT (3-(4.5-dimethylthiazol- 2yl)-2.5-diphenyltetrazolium bromide) assay. Results indicated that the *n*-hexanic extract from *A. unedo* leaves exhibited profound and highly significant inhibitory effect against *L. infantum* (IC_50_ = 64.05 ± 1.44 *µ*/mL) and *L. tropica* (IC_50_ = 79.57 ± 2.66 µg/mL) [93]. The authors suggested that *A. unedo* could constitute a potential source for antileishmanial agents. However, further research is needed to identify the bioactive compounds that are responsible for the antileishmanial activity and to determine their modes of action.

### 7.6. Anticancer and Cytotoxic Activity

The anticancer activity of methanolic, *n*-hexanic, and ethanolic extract from five medicinal plants including *A. unedo* was investigated *in vitro* against different cancer cell lines (L20B, RD and Vero) using the MTT assay. The pharmacological selectivity index (PSI) was calculated using PBMC as the normal cell line. It has been found that the n-hexanic extract of *A. unedo* leaves significantly inhibited the proliferation of RD (IC_50_ = 27.83 ± 1.20 *μ*g/mL; PSI = 0.69), L20 B (IC_50_ = 25.32 ± 1.26 *μ*g/mL; PSI = 0.76), and Vero cell lines (IC_50_ = 18.37 ± 1.23 *µ*g/ml; PSI = 1.05) [94]. As there are limited studies on the anticancer activity of the *A. unedo* extract, furthermore, in vitro as well as in vivo investigations are required to consolidate the role of *A. unedo* extract as a potential anticancerous drug. In addition, most of researchers focused on the whole crude extracts of *A. unedo* instead of the bioactive components with molecular mechanisms mostly unclear.

### 7.7. Antilithiasis Activity

Urolithiasis is a pathological problem that involves the formation of crystalline aggregates called “urinary stones” in the kidneys or in the urinary tract. The etiology of this disorder is related to a variety of metabolic and environmental disturbances [95]. Until now, there has been no satisfactory treatment for urolithiasis. Furthermore, medical management of urolithiasis can lead to side effects, which are sometimes very serious [96]. Thus, several medicinal plants have been used for the treatment and/or prevention of urolithiasis [97, 98].

Little information was recorded with the antilithiasic activity of *A. unedo*. A study by Kachkoul and coworkers assessed the effects of *A. unedo* leaf extracts (aqueous and hydroalcoholic extracts) on calcium oxalate crystallization *in vitro*. It has been found that the aqueous extract was more effective in the dissolution of calcium oxalate stones than the hydroalcoholic extract with dissolution values of 31.05% and 14.55%, respectively [62]. Another *in vitro* study on calcium oxalate crystallization has been performed with aqueous and hydroalcoholic extracts from *A. unedo* leaves using UV-visible spectrophotometric method. Results showed a higher potency of the plant aqueous extract compared to the hydroalcoholic extract against crystallization or nucleation at percentages of 69.41 ± 0.24 or 19.76 ± 0.27% and at 93.92 ± 2.61 and 45.16 ± 3.06% against the aggregation for both the aqueous and the hydroalcoholic extract, respectively [63]. This antilithiasic activity could be attributed to the high level of phenolic components contained in the leaves [63], which are known for their anticrystallizing property according to studies of both human urine as well as on animal models [99]. The authors concluded that *A. unedo* is a promising and effective remedy against “urinary stones”.

In a recent study, Baddade and colleagues investigated the antilithiasic activity of *A. unedo* fruits *in vitro*. Fruit samples were collected from six zones from the Beni Mellal-Khenifra region, and the antilithiasic activity was evaluated against the aggregation of calcium oxalate. Results showed that the fruit seed aqueous extract (3 mg/ml) inhibited the crystallization of calcium oxalate *in vitro*. At 10 mg/ml, the extract showed significant dissociation of the aggregates [100]. The authors suggested that *A. unedo* extracts seem to be promising against the crystallization of calcium oxalate, especially in the aggregation and nucleation stages.

## 8. Toxicological Properties

According to different toxicity tests, the use of *A. unedo* is devoid of any significant side effects and/or toxicity. Indeed, in an acute toxicity test performed in mice, we have demonstrated that the oral administration of *A. unedo* leaf aqueous extract at a dose up to 1200 mg/kg caused no mortality and no signs of adverse effects, demonstrating that the LD_50_ value of the *A. unedo* aqueous extract was higher than 1200 mg/kg b.w in oral administration in mice [44]. In another acute toxicity test realized in mice, it has been reported that the *A. unedo* root aqueous extract exhibited LD_50_ higher than 2000 mg/kg b.w with no adverse effect of this dose shown after single oral administration in mice [76]. Furthermore, in a study to analyze the acute toxicity of *A. unedo* and to evaluate its safety, an aqueous extract from *A. unedo* roots was intraperitoneally administrated in mice. The results did not show visible signs of toxicity or adverse effects even at a dose of 6 g/kg b.w [41].

The cytotoxicity of rat peritoneal macrophages treated with the ethanolic extract of *A. unedo* leaves was measured by the neutral red uptake assay. Results showed that rat peritoneal macrophages treated with the extract (6.02 ± 0.76 mg/ml) were able to uptake the red neutral dye after 3 days of incubation. It is concluded that the extract used at 6.02 ± 0.76 mg/ml had no cytotoxic effect [90]. In a previous study, we have examined the cytotoxic effect of *A. unedo* on platelet cell viability using calcein and trypan blue. Results showed that the incubation of human platelets with *A. unedo* leaf aqueous extracts (1.5 mg/ml) did not affect their viability [37].

## 9. Discussion

In the current review, we report the traditional medicinal uses and phytochemical and pharmacological properties of *A. unedo* from Morocco. Various *A. unedo* extract preparations have been broadly used in folk medicine for the management of several ailments. Different parts of the plant are used in the prevention and treatment of several complications, especially microbial infection, diabetes, hypertension, and gastrointestinal and renal diseases. In contrast, limited studies have been devoted to the plant fruits, although they represent an abundant source of phytochemicals with potent antioxidant capacity. Different traditional uses have been confirmed by pharmacological activities of the plant extracts and its identified compounds using different animal models *in vitro* and *in vivo*. Among the bioactive compounds, flavonoids and tannins are the most important, being responsible for most of the pharmacological activities reported, namely, antioxidant, antiaggregant, antidiabetic, antihypertensive, antimicrobial, anticancer, or antidiarrheal activities. These activities generally agree with traditional knowledge and folk medicine.

The studied *A. unedo* extracts have been shown to be a rich source of epicatechin, catechin, catechin gallate, flavonoids, anthocyanins and tannins, and phytochemicals with well-recognized antioxidant capacity. The antioxidant properties of *A. unedo* extracts have been evidenced in different *in vitro* systems and *in vivo* studies in rats, mice, and cell culture systems. The antihypertensive, anti-platelet, anticancer, and antidiabetic properties of the plant are all believed to be related to its antioxidant activity, although no direct associations have been made.

Various studies involving catechin, epicatechin, tannins, anthocyanins, and flavonoid-rich extracts from other plants have demonstrated antiaggregant [101–104], antidiabetic [105], anticancer [106], and vasodilation properties [107]. Therefore, it is not surprising that various *A. unedo* extracts exhibit these properties due to the presence of catechins, anthocyanins, and flavonoids. Significant variations were observed in phytochemical composition among investigated *A. unedo* extracts, which are mainly due to genetic variability and environmental factors, such as bioclimatic or geographical origin [39, 54, 55, 64]. Furthermore, it was revealed that the biological properties of *A. unedo* depend on the extraction method, the nature of phytochemicals, and their levels in the plant extracts.

The retrieved studies were insufficient to substantiate all the traditional medicinal claims of the plant. Moreover, most of the studies that highlighted the beneficial properties of *A. unedo* on health were *in vitro* and *in vivo* investigations on animal models as well as several cell lines, which represent one of the limitations in this review. Thus, more clinical studies are necessary to validate the traditional uses and pharmacological activities of the selected plant.

In addition to details regarding the traditional use of *A. unedo*, this review also focuses on the nutritional characteristics of the plant and its potential uses for other industries, such as food, cosmetics, or pharmaceutical ones, especially the fruit, which constitutes an important source of compounds with beneficial health properties such as minerals, vitamins, fiber, flavonoids, and tannins.

Different parts of the plant have been used for a long time without any toxicity or side effects. In addition, *A. unedo* fruits have been consumed for many years in folk medicine due to its medicinal properties and nutritional value with no known toxicity. Furthermore, toxicity tests carried out on animals or *in vitro* did not demonstrate any toxic effects on the plant. However, we note the absence of long-term studies either on animals or on humans that could address its long-term safety. Thus, unlike pharmaceutical drugs, *A. unedo* not only has no side effects, but has various medicinal properties against several diseases.

## 10. Conclusion

Numerous studies have reported that *A. unedo* is an important source of catechins, tannins, anthocyanins, as well as flavonoids with potent antioxidant capacity. The antioxidant properties of the plant have been well demonstrated through both *in vitro* and *in vivo* investigations. The antioxidant activity of the plant seems to be responsible for the antidiabetic, anticancer, antiaggregant, and antihypertensive activities that have been demonstrated. However, the majority of the phytochemical investigations was limited to screening studies rather than the isolation of bioactive constituents. In addition, several researchers focused on the whole crude extracts of *A. unedo* instead of the bioactive components, with molecular mechanisms mostly unclear. Additionally, no human clinical studies have been reported. In light of this, relevant goals for future research are the identification of bioactive molecules from *A. unedo* using bioguided isolation assays and finding evidence for their beneficial health effects through preclinical and clinical studies. The physical properties of the equipment used in the transport, storage, harvesting, and processing of *A. unedo* fruits must also be investigated.

## Figures and Tables

**Table 1:** Traditional uses of *A. unedo* L. in Morocco.

Part used	Traditional medicinal uses	Mode of preparation/administration	References
Leaves	Kidney diseases	Decoction	[24]
	Antidiabetic, antihypertensive	Decoction/infusion	[22, 108]
	Cardiac disease, diabetes, hypertension	N.D.	[23]
	Antidiabetic, astringent, antidiarrheal	Decoction	[25, 26, 61]
	Stomachic, antiasthmatic	Decoction	[27]

Roots	Antidiabetic, antihypertensive, anti-inflammatory, against rheumatism	Decoction/infusion	[22, 25, 108]
	Cardiac disease, diabetes	N.D.	[23]
	Cholesterol-lowering, digestive problems, cardiovascular diseases	Decoction	[60]
	Diabetes	Decoction	[26]
	Stomachic, antiasthmatic	Decoction	[20, 27]

Fruits	Kidney diseases	Raw	[24]
	Digestive diseases and diarrhea	Infusion, fresh fruit	[19, 21]

Bark	Cholesterol-lowering, digestive problems, cardiovascular diseases	Decoction	[60]
	Diuretic, astringent, antidiarrheal	Decoction	[25]

N.D: not determined.

**Table 2:** Major phytochemical compounds reported from A. unedo *L*.

Plant part	Major components	Extraction method	References
Leaves	Genins and heterosidic flavonoids	Solid-liquid extraction	[37]
		Colorimetric method	
		Soxhlet extraction method	
	Tannins	Soxhlet extraction method	[47, 48]
	Arbutoside, quercetin, epicatechin, catechin, catechin gallate, hyperoside and gallic acid	HPLC coupled with mass spectrometry	[47]
	Tannins, flavonoids, anthraquinones, terpenoids, free quinones	Colorimetric method	[67]
	Polyphenols, flavonoids, tannins.	Infusion	[63]
		Soxhlet extraction method	
		Colorimetric method	
	Phenolic compounds, flavonoids, flavonols, and anthocyanins	Colorimetric method and UPLC-PDA-ESI-MS	[62]
	Gallic acid derivatives and flavonoids (22 compounds)		

Roots	Tannins, flavonoids, anthraquinones, terpenoids, free quinones	Colorimetric method	[67]
	Catechin	XAD-16 resin	[50]
		HPLC	

Fruit	Flavonoids, tannins, anthocyanins, anthraquinones, sterols, steroids, deoxysugars, and glycosides	Colorimetric method	[65]
	75 compounds identified as hydroxybenzoic acids, hydroxycinnamic acids, flavone, flavan-3-ols, flavonols, and dihydroflavonols.	HPLC	
		UPLC-PDA-ESI-MS	
	Phenols, flavonoids, condensed tannins, hydrolyzable tannins, and anthocyanins.	Spectrophotometric methods	[66]
	17 phenolic compounds, of which gallocatechol and catechin were the most abundant	HPLC	

UPLC-PDA-ESI-MS, Ultra-performance liquid chromatography with photodiode array and electrospray ionization tandem mass spectrometry; HPLC, high-performance liquid chromatography.

**Table 3:** Pharmacological activities of A. unedo *L*.

Pharmacological effect	Plant part	Extract/fraction	Model applied	Effect/mechanism(s) of action	References
Antidiabetic activity	Roots	Water	OGTT	Antihyperglycemic effect	[41]
			IVGTT	Inhibition of jejunal glucose absorption	
	Roots	Water	OGTT	Hypoglycemic effect in n-stz induced-diabetic rats (*in vivo*)	[46]
			n-stz-induced diabetic rats (*in vivo*)	Potentiation of the insulin activity	
				Improved glucose peripheral consumption	
	Roots	Catechin	*α*-glucosidase assay	Antidiabetic effect through inhibition of *α*-glucosidase enzyme	[50]
	Roots	Water	*α*-glucosidase and *α*-amylase assays	Inhibition of *α*-glucosidase and *α*-amylase	[76]
			STZ-NA-induced-diabetic mice (*in vivo*)	Regeneration of pancreatic *β*-cells	
	Roots bark	Water	OGTT	Inhibition of SGLT	[78]
			Short-circuit current technique (*in vitro*)	Improved oral glucose tolerance	

Antihypertensive and vasorelaxant activity	Roots and leaves	Water	*In vivo* determination of both blood pressure and baroreflex sensitivity. *Ex vivo* analysis of vascular reactivity.	Reduces the development of increased SBP	[42]
				Ameliorates vascular reactivity and baroreflex sensitivity	
				Normalizes renal function	
				Prevents the myocardial hypertrophy (roots extract)	
	Leaves	Aqueous extract soxhlet extraction: Hexane, dichloromethane, ethyl acetate, methanol and water	*In vitro* study of vasorelaxant effect	Endothelium-dependent vasorelaxant activity mediated by NO. This effect is due to the presence of condensed tannins and catechin gallate.	[47]
	Roots	Water extract	*In vitro* study of vasodilator effect and mechanisms of action	Endothelium-dependent relaxation of aorta mainly mediated by a stimulation of endothelial NO synthase	[43]
	Roots	Water	*In vivo* study of hypertension	Delayed the development of hypertension	[49]
			Measurement of diuresis	Attenuated the pressor responses to phenylephrine and angiotensin I diuretic effect	

Antiaggregant activity	Leaves	Genins (free flavonoids) heterosidic flavonoids	*In vitro* measurement of platelet aggregation	Inhibition of rat platelet aggregation induced by thrombin (effect mainly due to flavonoids)	[44]
	Leaves	Water Genins (free flavonoids) Heterosidic flavonoids	*In vitro* measurement of platelet aggregation	Inhibition of human platelet aggregation	[37]
				Attenuation of Ca^2+^ mobilization	
				Reduction of ROS production	
				Inhibition of protein tyrosine phosphorylation	
	Leaves	Water	*In vitro* measurement of platelet aggregation	Inhibition of rat platelet aggregation induced by thrombin (effect probably due to tannins)	[48]
		Petroleum ether			
		Dichloromethane			
		Ethyl acetate			
		Methanol			
		Tannins			
	Roots	Water	*In vitro* measurement of platelet aggregation	Inhibition of thrombin- and ADP-induced rat platelet aggregation	[45]

Antioxidant activity	Fruits	Phenolic	ABTS assay	Radical scavenging activity (ABTS)	[66]
		Flavonoids		Antioxidant capacity varies with the genotypes and variations in plants chemical composition	
		Condensed tannins			
		Hydrolyzable tannins			
		Anthocyanins			
	Leaves	Water	DPPH and FRAP assays	Radical scavenging activity (DPPH and FRAP)	[62]
		Hydroalcoholic extractHydroalcoholic extract		Potent antioxidant activity with hydroalcoholic extract	
	Roots and leaves	Aqueous extract	DPPH assay	In vitro antioxidant activity in DPPH assay	[67]
			*In vivo* antioxidant assay (dosage of MDA and SOD)	*In vivo* antioxidant activity	
				Roots exhibited better activity than leaves	
	Fruits from three different regions (AA, AC and AQ)	Ethyl acetate MeOH/water 80 : 20 (v/v)	DPPH assay	Antiradical ability of all *A. unedo* samples	[65]
				Higher antioxidant activity with samples extracted by MeOH:water	
				Higher antioxidant activity with AC fruits	
				Antioxidant activity depends on the geographic origin of fruit and its phenolic content	
	Leaves	Ethyl acetate, ethanol, methanol, and n-hexane	DPPH assay	Ability to act as DPPH radicals scavenger for all the extracts	[87]
				n-Hexane and methanol extracts showed the highest radical scavenging activity	
				The antioxidant activity is correlated with phytochemical content	

Antibacterial and antiparasitic activity	Leaves	Ethyl acetate, ethanol, methanol, and *n*-hexane.	Determination of MIC by the dilution agar method.	Antibacterial activity against gram-negative (*Escherichia coli, Pseudomonas aeruginosa)* and gram-positive (*Staphylococcus aureus, Listeria monocytogenes*) bacteria	[87]
			Agar well diffusion assay		
	Leaves	Water	Determination of growth inhibition values by paper disc	Antimycobacterial activity against *Mycobacterium bovis, Mycobacterium smegmatis and Mycobacterium aurum*	[90]
		Ethanol			
	Leaves	*n*-hexane, methanol, and ethanol	MTT assay	Antileishmanial activity using *Leishmania major, Leishmania tropica, and Leishmania infantum*	[93]

Anticancer and cytotoxic activity	Leaves	*n*-hexane, methanol, and ethanol	MTT assay	Cytotoxic effects against L20 B, RD and vero cell lines with *A. unedo n*-hexane extract	[94]
			Cancerous cell lines: L20B, RD and vero		
			Normal cells: PBMC		
	Leaves	Water	Cell viability evaluated using calcein and trypan blue.	No toxic effect was observed in human platelets cell viability with a concentration of 1.5 mg/mL	[37]
	Leaves	Ethanol	Neutral red uptake assay	No toxic effect was observed in peritoneal macrophages with a concentration of 6.02 ± 0.76 mg/mL	[90]

Antilithiasic activity	Leaves	Water	Measurement of litholytic activity using a model structure resembling the urinary circuit	Litholytic activity against calcium oxalate stones	[62]
		Hydroalcoholic extract		Higher litholytic activity with aqueous extract	
	Leaves	Water Hydroalcoholic extract	*In vitro* measurement of the crystal formation by using a UV-Visible spectrophotometer	Inhibitory activity against calcium oxalate crystallization	[63]
				Higher litholytic activity with aqueous extract	
			Microscopic observation of the crystals	This effect is due to the presence of polar compounds in the plant extracts such as polyphenols	
	Fruits (seeds)	Water	Polarizing optical microscope (PLM)	Inhibition of the crystallization of calcium oxalate	[100]

OGTT, oral glucose tolerance test; n-stz, neonatal streptozotocin; IVGTT, intravenous glucose tolerance test; STZ-NA, streptozotocin-nicotinamide; SGLT, sodium-dependent glucose transporter; SBP, systolic blood pressure; NO, nitric oxide; Ca2+, calcium; ROS, reactive oxygen species; ABTS, 2,20-azinobis-(3-ethylbenzothiazoline-6-sulfonic acid); DPPH, 1,1-diphenyl-2-picrylhydrazyl; FRAP, ferric reducing/antioxidant power; ADP, adenosine diphosphate; SOD, superoxide dismutase; MDA, malondialdehyde; AA, achakar; AQ, qsar Kbir; AC, chaoun-Qalaa; MIC, minimal inhibitory concentration; MTT, (3-(4,5-dimethylthiazol-2-yl)-2,5-diphenyl tetrazolium bromide).

## Data Availability

The data supporting this review were taken from previously reported studies and datasets, which have been cited. The processed data are available from the corresponding author upon request.

## Abbreviations

WHO: World health organization
DM: Diabetes mellitus
A. unedo*:*: Arbutus unedo
B.W: Body weight
Ca^2+^: Calcium
IC_50_: Median inhibitory concentration
D.W: Dry weight
ROS: Reactive oxygen species
CVD: Cardiovascular diseases.
